# Management of Fragility Femoral Fractures: A UK Regional Multicentre Audit of the British Orthopaedic Association Standards for Trauma (BOAST) Guidelines

**DOI:** 10.7759/cureus.109830

**Published:** 2026-05-28

**Authors:** Rory Ormiston, Kim Pearce, Daniel Hancock, Suresh Kondi, Ahmed Abdellatif, Sophie White, Tom Moore, Tara Edwards, Zoe Lyon, Ali Assaf, Alex Denning

**Affiliations:** 1 Trauma and Orthopaedics, University Hospital Southampton NHS Foundation Trust, Southampton, GBR; 2 Trauma and Orthopaedics, University Hospitals Dorset NHS Foundation Trust, Poole, GBR; 3 Trauma and Orthopaedics, Portsmouth Hospitals University NHS Trust, Portsmouth, GBR; 4 Trauma and Orthopaedics, Isle of Wight NHS Trust, Newport, GBR; 5 Trauma and Orthopaedics, Dorset County Hospital NHS Foundation Trust, Dorchester, GBR; 6 Trauma and Orthopaedics, Hampshire Hospitals NHS Foundation Trust, Basingstoke, GBR; 7 Trauma and Orthopaedics, Salisbury NHS Foundation Trust, Salisbury, GBR

**Keywords:** boast, femur, fragility, length of stay, weight-bearing status

## Abstract

Background

Fragility fractures of the femur, excluding the neck of the femur (NOF), are increasingly common and associated with significant morbidity in the older population. Current best practice in the UK recommends that surgery should permit full weight bearing (FWB) and be performed within 36 hours of admission.

Methods

A multicentre retrospective audit was conducted across patients admitted to seven NHS hospitals in the UK in 2021. Patients aged ≥65 years with low-energy, non-NOF femoral fragility fractures who underwent surgery were included. Data were collected on fracture classification, operative treatment, weight-bearing prescription and achievement, time to surgery, length of stay, and discharge destination.

Results

A total of 358 patients met the inclusion criteria: hip periprosthetic fractures (n = 171, 48%), distal femur fractures (n = 67, 19%), midshaft fractures (n = 65, 18%), and knee periprosthetic fractures (n = 55, 15%). Fixation was the predominant treatment (n = 303, 85%), with plating being the most common. Joint arthroplasty (n = 54, 15%) was associated with more permissive postoperative prescriptions than fixation (n = 41 (76%) vs. n = 173 (57%)). Of 342 patients with time-to-surgery data, only 86 (25%) received surgery within 36 hours. Arthroplasty procedures more frequently breached this timeframe (n = 46, 85%) when compared to fixation (n = 218, 72%). Median length of stay was shorter when surgery occurred within 36 hours (13 days vs. 17 days). Weight-bearing prescription was not associated with length of stay, but was associated with discharge destination. FWB patients were more frequently discharged home.

Conclusion

In the UK, fixation remains the most common treatment for non-NOF fragility femoral fractures, despite being associated with less permissive weight-bearing prescriptions and less independent discharge outcomes than arthroplasty. Delay beyond 36 hours was common, particularly in arthroplasty cases, and was associated with prolonged hospital stay. Improved access to revision arthroplasty surgeons is required to achieve compliance with the British Orthopaedic Association Standards for Trauma (BOAST) guidance.

## Introduction

In the UK, fragility fractures of the femur are common. Fractures at the neck of the femur (NOF) have the highest incidence, with over 70,000 cases per year [1]. The standard of care in this population is well defined and continually audited by the National Hip Fracture Database (NHFD). In 2020, the NHFD implemented an extended scope to include other femoral fragility fractures. This reflects the complexity of these fractures and the burden they represent to the individual and the healthcare system. The 2025 NHFD Annual Report documented 5,096 peri-prosthetic fractures, 1,922 distal femoral fractures, and 1,271 midshaft femoral fractures [1].

The number of peri-prosthetic fractures is likely to rise in keeping with the increasing number of primary hip and knee arthroplasties being performed in the UK. With approximately 100,000 of each of total knee arthroplasty and total hip arthroplasty being performed in 2018, these figures are projected to rise to approximately 130,000 each by 2060 [2].

The treatment of non-NOF femoral fragility fractures is multifactorial, including orthogeriatric, bone health, and nutritional assessments, prolonged physiotherapy, and often additional support is required on discharge. Selecting the appropriate surgical approach is critical to initiating a successful multifactorial recovery.

The British Orthopaedic Association Standards for Trauma (BOAST) process guideline ‘The care of the older or frail orthopaedic trauma patient’ was published in 2019 [3]. This recommended offering the same multidisciplinary care received by NOF patients to all femoral fragility fracture patients. There is an explicit recommendation that all surgery in this population should be performed to allow full weight bearing (FWB). Similar guidance from the National Institute for Health and Care Excellence (NICE) has been in place since 2016. NICE guideline 38 recommends unrestricted weight bearing after surgery for the treatment of distal femoral fractures [4].

Some variation in practice may arise from anxiety around stability, especially in this population with osteoporotic bone treated with open reduction and internal fixation [5]. To support this concern, cadaveric biomechanical analysis has shown plastic deformity with plate fixation, and the authors recommend against immediate weight bearing [6]. These biomechanical data are supported by clinical evidence, which acts to discourage early FWB. A recent pilot study of distal femoral fractures treated with plate fixation demonstrated no difference in outcomes associated with early or non-weight-bearing groups [7]. These factors underpin the hesitation to allow immediate FWB after fixation in patients with osteoporotic bone.

This Wessex regional audit identified patients with non-NOF fragility fractures of the femur and investigated compliance with the BOAST guidelines. The BOAST guidelines are broad; thus, the specific objectives of this study were to identify the surgical strategies used, the prescribed weight-bearing status, and the time to surgery achieved.

## Materials and methods

Study design

A retrospective observational audit was conducted across seven NHS hospitals in the Wessex Deanery, including Southampton, Portsmouth, Poole, Salisbury, Basingstoke, Dorchester, and the Isle of Wight. The audit was against the BOAST guidelines for 'The care of the older or frail orthopaedic trauma patient' [3], specifically point 14, i.e., all surgery in the frail patient should be performed to allow full weight bearing for activities required for daily living and within 36 hours of admission, in line with current hip fracture care.

Each centre received institutional audit approval. Patients admitted in 2021 and meeting the inclusion criteria were included.

The inclusion criteria were as follows: patients must be aged ≥65 years old, must have sustained an injury through a low-energy mechanism, the injury must be a non-NOF femoral fragility fracture (including periprosthetic fractures, distal femoral fractures, midshaft femoral fractures), and must have undergone surgical treatment.

The exclusion criteria were any patients under 65 years old, sustaining high-energy injuries and/or polytrauma of the affected limb, and those patients who did not have surgical management.

The primary outcome was to audit the prescribed and achieved weight-bearing status for these patients following surgical treatment. Secondary outcomes included time between admission and surgery, length of stay, and discharge destination.

Case identification and data sources

Patients meeting the inclusion criteria were identified through theatre logbooks and the hospital coding database. Data were collected retrospectively from each hospital’s electronic health record system and supplemented by paper-based documentation where necessary. For each eligible patient, a detailed review was undertaken of Picture Archiving and Communication System (PACS) images, operation records, ward progress notes, and discharge summaries covering the entire inpatient stay.

Fracture classification included site-specific systems, with the Vancouver classification applied to all total hip arthroplasty (THA) periprosthetic fractures and the Su classification applied to all total knee arthroplasty (TKA) periprosthetic fractures.

Data management and analysis

All collected data were anonymised and entered into a secure, password-protected spreadsheet for analysis.

Data collection occurred before the British Orthopaedic Association (BOA) standardisation of weight-bearing terminology. There was a large variation in wording of post-operative weight-bearing prescriptions, which were simplified into FWB (including weight bearing as able), partial weight bearing (PWB) (including all toe touch weight bearing, weight bear with frame) and non-weight bearing (NWB) (including transfer only).

Whether the prescribed weight-bearing status was achieved was recorded by doctors at each site. There was no specific definition or timeframe for this assessment, and a context-specific judgment was made by each assessing doctor during data capture.

Data analysis and graph creation were completed in Python (Python Software Foundation, Wilmington, Delaware) using pandas for data handling and matplotlib for graphing.

## Results

**Figure 1:**
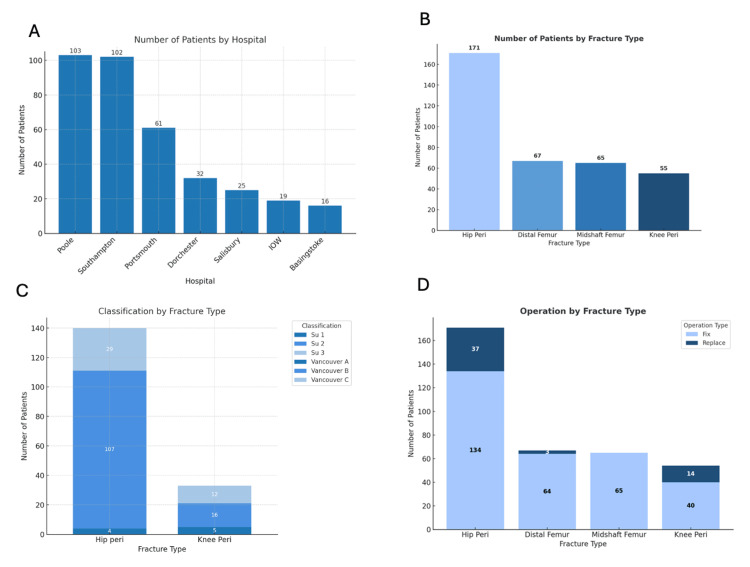
Descriptive analytics. (A) Number of patients by hospital. (B) Number of patients by fracture type. (C) Classification by fracture type. (D) Operation by fracture type. IOW: Isle of Wight.

A total of 358 patients were included in the analysis across the seven hospitals. The majority of patients were treated at Poole (n = 103), followed by Southampton (n = 102), Portsmouth (n = 61), Dorchester (n = 32), Salisbury (n = 25), Isle of Wight (n = 19), and Basingstoke (n = 16) (Figure 1A). The most common fracture types were hip periprosthetic (n = 171), distal femur (n = 67), midshaft femur (n = 65) and knee periprosthetic (n = 55) (Figure 1B). The breakdown of peri-prosthetic fracture classification can be seen in Figure 1C.

Fixation strategies

Of the total cohort, 303 underwent fixation procedures, and 54 underwent arthroplasty procedures. One patient was treated with amputation, which is out of the scope of this study and will be excluded from further analysis. Fixation methods included plating (n = 206), intramedullary nailing (n = 79), nail/plate combination (n = 12), screw fixation (n = 3), cable fixation (n = 2), and external fixation (n = 1). Joint arthroplasty operations included hip revision (n = 37), knee revision (n = 10), and distal femur arthroplasty (n = 7). The number of patients who underwent fixation vs. arthroplasty per fracture type can be seen in Figure 1D.

**Figure 2:**
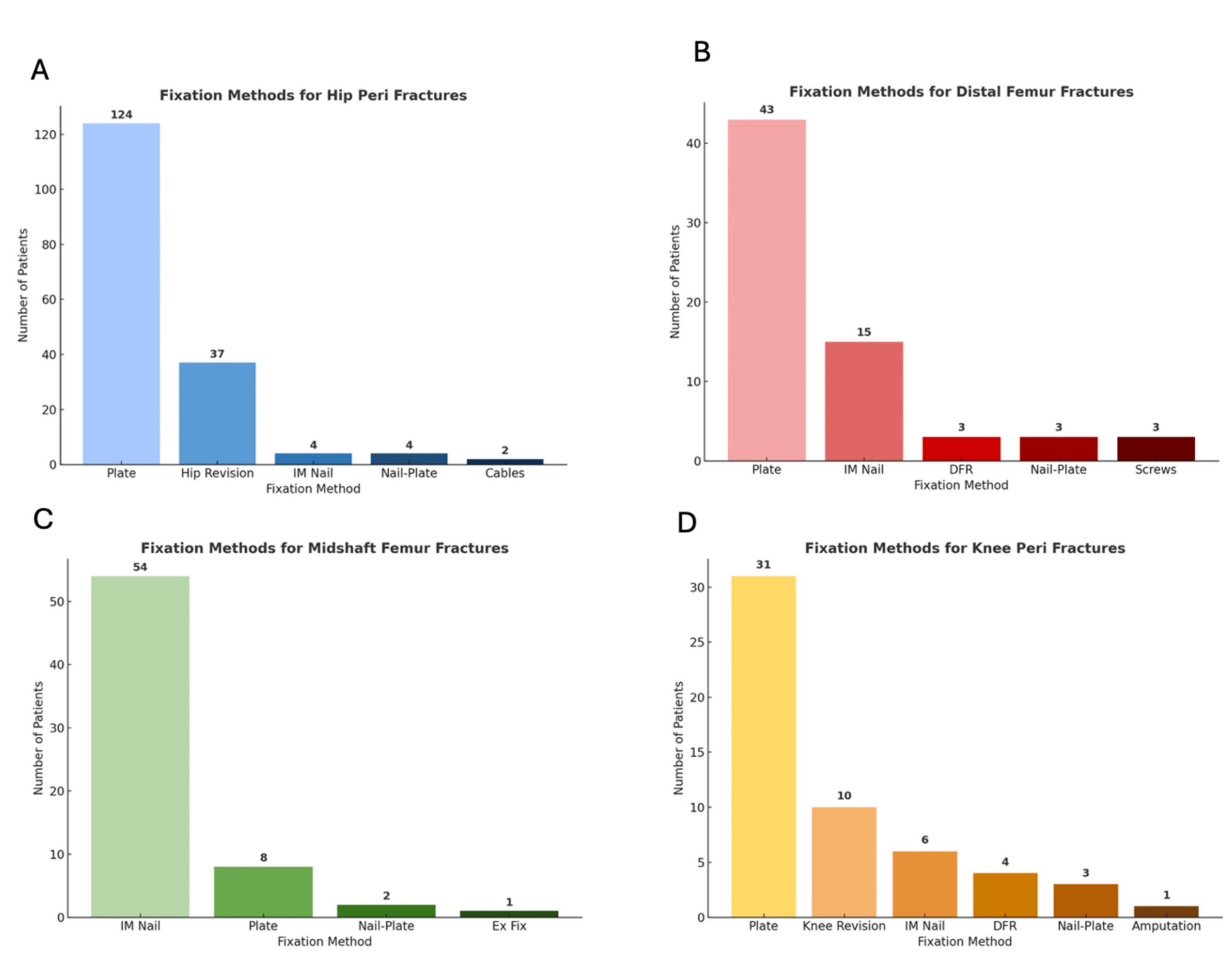
Fixation method and fracture type. (A) Fixation methods for hip periprosthetic fractures. (B) Fixation methods for distal femur fractures. (C) Fixation methods for midshaft femur fractures. (D) Fixation methods for knee periprosthetic fractures. DFR: distal femoral replacement; IM Nail: intramedullary nail; Ex Fix: external fixation.

Fracture type was closely associated with the type of operation performed. The breakdown of fracture type and operative treatment can be seen in Table 1 and Figure 2.

**Table 1:** Fracture type and operative treatment. DFR: distal femoral replacement; IM Nail: intramedullary nail; Ex Fix: external fixation.

Fracture type	Plate	IM Nail	Nail-Plate	DFR	Hip revision	Knee revision	Cables only	Ex Fix	Screws only	Amputation	Total
Hip periprosthetic	124	4	4	0	37	0	2	0	0	0	171
Distal femur	43	15	3	3	0	0	0	0	3	0	67
Midshaft femur	8	54	2	0	0	0	0	1	0	0	65
Knee periprosthetic	31	6	3	4	0	10	0	0	0	1	55
Total	206	79	12	7	37	10	2	1	0	1	358

Weight-bearing prescriptions

**Figure 3:**
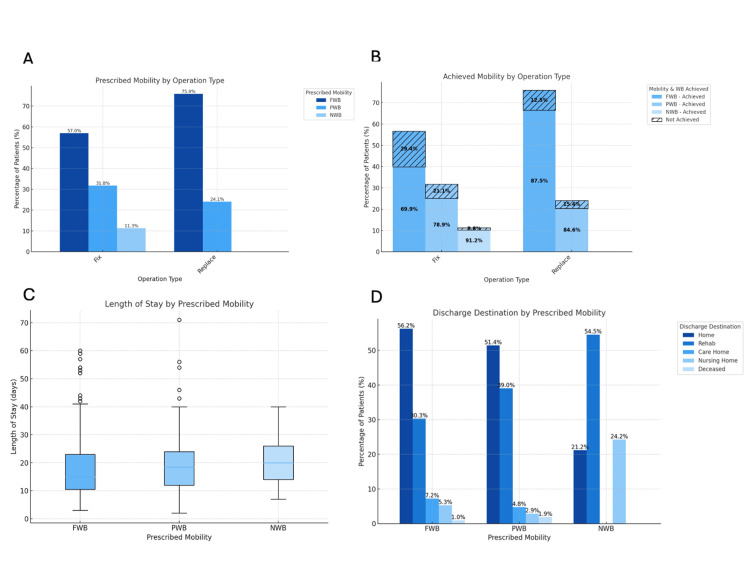
Prescribed mobility. (A) Prescribed mobility by operation type. (B) Achieved mobility by operation type – hashed out areas of each bar represent those patients who did not achieve their prescribed weight-bearing status. (C) Length of stay by prescribed mobility status. (D) Discharge destination by prescribed mobility status. FWB: full weight bearing; PWB: partial weight bearing; NWB: non-weight bearing.

Prescribed mobility varied according to operation type; patients receiving joint arthroplasty surgery were prescribed FWB more frequently than patients receiving fixation (n = 41 (76%) vs. n = 173 (57%)), and patients receiving arthroplasty surgery were never prescribed a NWB status (Figure 3A). Prescribed weight-bearing status did not always correlate with achieved weight-bearing status. Figure 3B demonstrates that patients prescribed FWB more were more likely to achieve this who received arthroplasty than fixation (n = 36 (87%) vs. n = 121 (70%)). No significant relationship was observed between prescribed mobility and length of stay (Figure 3C), but a marginally higher proportion of patients prescribed FWB were discharged home compared to those prescribed PWB and considerably more than those prescribed NWB (n = 120 (56%) vs. n = 56 (51%) vs. n = 7 (21%)) (Figure 3D). Similarly, NWB patients are far more likely to be discharged to a rehab establishment or nursing home (Figure 3D).

A subgroup analysis was conducted of the 226 periprosthetic fractures. Subgroup analysis found trends consistent with the whole dataset. Patients receiving arthroplasty were associated with more permissive weight-bearing prescriptions, which were in turn associated with a more independent discharge destination but with no observable difference in length of stay.

Time to surgery

**Figure 4:**
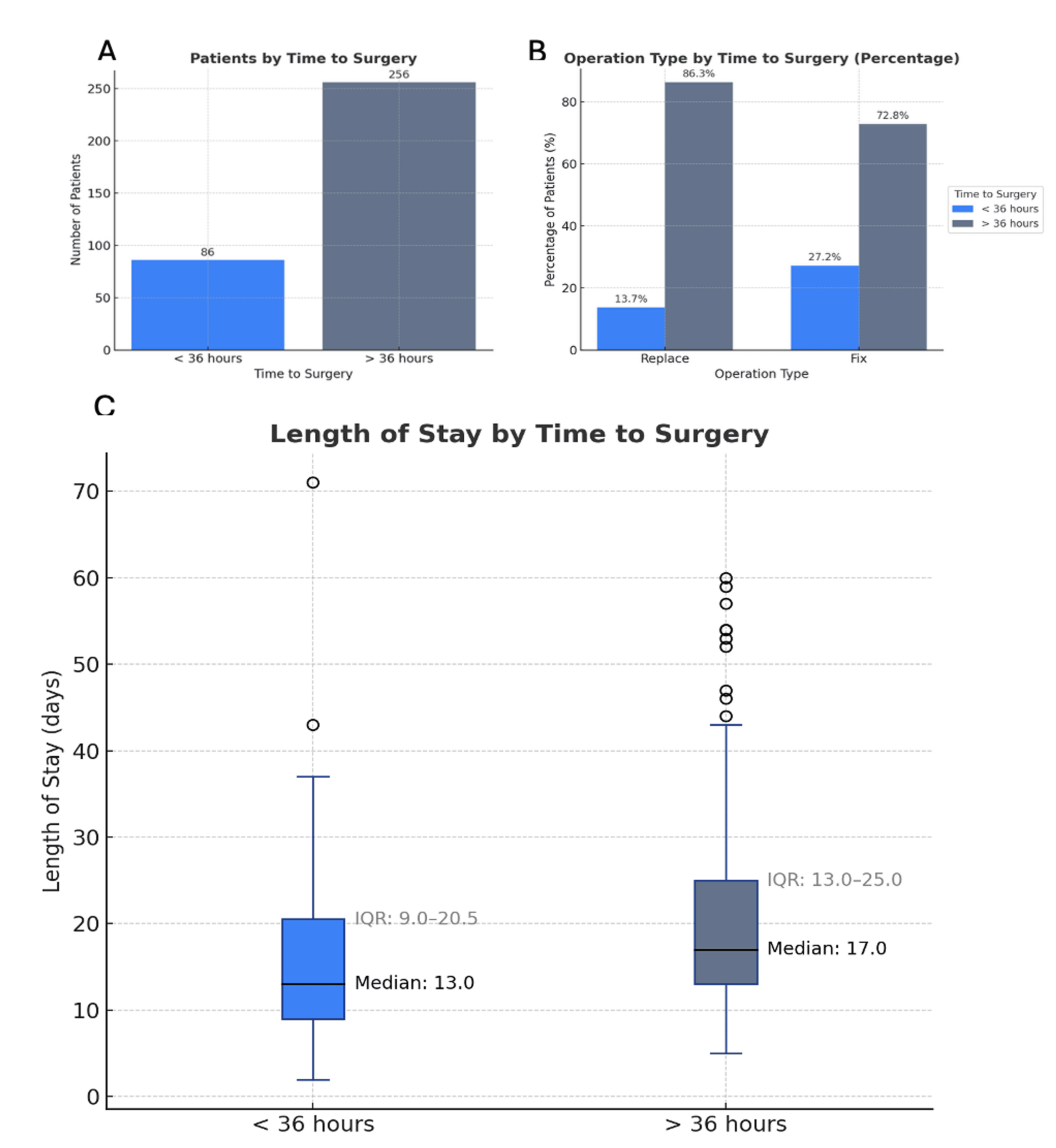
Time to surgery analysis. (A) Patients by time to surgery. (B) Operation type by time to surgery as a percentage. (C) Length of stay by time to surgery.

Of the total 358 patients, 342 had data collected on the length of time between admission and surgery; patients missing these data were excluded from further analysis, and no data imputation was performed. A total of 86 patients received surgery within the BOAST 36-hour recommended timeframe; the remaining 256 patients had surgery more than 36 hours after admission (Figure 4A). Patients receiving joint arthroplasty were more likely to wait over 36 hours for surgery than those receiving fixation (n = 46 (85%) vs. n = 218 (72%)) (Figure 4B). Median length of stay was lower in patients who received surgical treatment within 36 hours (13 days vs. 17 days) (Figure 4C).

## Discussion

This multicentre regional audit reports findings that are consistent with existing literature demonstrating that non-NOF femoral fragility fractures represent a significant and growing clinical burden in an ageing population, with outcomes comparable to those seen in hip fracture cohorts [8].

Despite concerns about allowing FWB after fixation [5-7], this remains the most common weight-bearing prescription for all fracture types in this study. During data capture, considerable variation in wording for postoperative weight-bearing prescriptions created challenges in categorisation and analysis. The authors note that these data were collected before the 2024 BOAST guidance on specific weight-bearing terminology and prescriptions [9], which would help to clarify categorisation and analysis in future work. Despite this, there is an observed difference between surgeon-prescribed and patient-achieved weight-bearing status. Weight-bearing status is a multifactorial challenge dependent not only on the type of surgery but also on post-operative complications, delirium, pain, analgesia, physiotherapy input, mobility aids, and patient motivation. It is likely that patients in both FWB and PWB groups self-regulate their weight bearing to a degree.

The existing literature surrounding postoperative weight bearing remains mixed. National guidance from the National Institute for Health and Care Excellence recommends unrestricted weight bearing following surgery for distal femoral fractures [4], reflecting the functional and systemic risks associated with immobility in this population. However, concerns persist regarding fixation stability in osteoporotic bone, particularly following locked plate constructs [5,6]. More recent clinical evidence has challenged this paradigm. For example, Paulsson et al. demonstrated that immediate FWB following plate fixation of distal femoral fractures in elderly patients did not result in increased complications compared with restricted weight bearing [10]. Similarly, systematic review data suggest that early weight bearing after distal femoral fracture fixation may be safe in selected patients [11]. These findings support the observation in this audit that FWB is frequently prescribed even after fixation, although a substantial proportion of patients do not achieve their prescribed mobility.

Patients receiving arthroplasty, rather than fixation, were associated with more permissive weight-bearing prescriptions, with no arthroplasty patients being prescribed a NWB status. This finding aligns with the literature, where arthroplasty, particularly distal femoral replacement or revision hip arthroplasty, is often advocated in frail patients to facilitate immediate mobilisation and reduce dependence on fracture healing [12,13]. However, arthroplasty procedures are resource-intensive, requiring specialist surgeons, implants, and theatre availability. This likely explains the finding in this audit that patients undergoing arthroplasty were more likely to experience delays to surgery beyond 36 hours.

Weight-bearing prescription was not associated with length of stay in this study, but was associated with discharge destination. A more permissive weight-bearing prescription was associated with discharge to a more independent level of care. This supports existing evidence that early mobilisation is a key determinant of functional recovery and discharge planning in frail orthopaedic populations [8].

Patients who are operated on within 36 hours of admission have a shorter length of stay. However, only a minority of patients in this cohort achieved this target. Patients requiring arthroplasty were more likely to experience delays, likely reflecting the logistical challenges described above. While the evidence base for time-to-surgery in non-NOF femoral fragility fractures is less well established than for hip fractures, some studies suggest that delays in surgical management of periprosthetic femoral fractures may increase morbidity and length of stay [14]. This aligns with the findings of this audit and reinforces the rationale for treating these injuries with similar urgency to hip fractures, as recommended by BOAST guidance.

The authors acknowledge several weaknesses of this study. This is a real-world pragmatic observational audit reporting on a heterogeneous group of fracture types and surgical approaches, which have been grouped on the basis that all patients should receive surgery that enables early FWB. As an observational study, this study is subject to confounding by indication. Further to this, it is not powered to infer statistical significance or causation between observed relationships. For example, the observed association between NWB status and discharge to institutional care may reflect underlying patient frailty rather than a direct causal relationship. Secondly, incomplete case capture at one site highlights potential limitations in data collection methodology. Finally, the lack of a specific definition for 'achieved weight-bearing status' means this is being reported as an in-context judgement assessment and should be interpreted as such.

This work has identified important trends between treatment strategies and outcomes, and provides a basis for hypothesis generation. In particular, the observed differences between fixation and arthroplasty in weight-bearing prescription and discharge outcomes highlight the need for higher-level evidence. A randomised controlled trial comparing fixation and arthroplasty in selected fragility distal femoral and periprosthetic fractures, where surgical equipoise exists, would help to clarify causative relationships and guide optimal management strategies.

## Conclusions

This UK-based, multicentre regional audit has shown that fixation is still the most observed surgical treatment option despite an association with less permissive weight-bearing prescriptions and a less independent discharge destination than arthroplasty surgery. This trend is possibly related to surgical anxiety regarding FWB after fixation and is against current national guidelines.

Early surgery within 36 hours is associated with shorter length of stay, but is often difficult to achieve for patients requiring joint arthroplasty, possibly due to the need for specialist revision arthroplasty surgeons and equipment. Improving access to these resources is essential to enable early FWB in accordance with BOAST guidelines.
